# Measurement of clinical documentation burden among physicians and nurses using electronic health records: a scoping review

**DOI:** 10.1093/jamia/ocaa325

**Published:** 2021-01-12

**Authors:** Amanda J Moy, Jessica M Schwartz, RuiJun Chen, Shirin Sadri, Eugene Lucas, Kenrick D Cato, Sarah Collins Rossetti

**Affiliations:** 1 Department of Biomedical Informatics, Columbia University, New York, New York, USA; 2 School of Nursing, Columbia University, New York, New York, USA; 3 Department of Translational Data Science and Informatics, Geisinger, Danville, Pennsylvania, USA; 4 Vagelos School of Physicians and Surgeons, Columbia University New York, New York, USA; 5 Department of Medicine, Weill Cornell Medical College, New York, New York, USA

**Keywords:** electronic health records, physicians, nurses, documentation, workflow, health policy, burden

## Abstract

**Background:**

**Objective:**

Electronic health records (EHRs) are linked with documentation burden resulting in clinician burnout. While clear classifications and validated measures of *burnout* exist, documentation *burden* remains ill-defined and inconsistently measured. We aim to conduct a scoping review focused on identifying approaches to documentation burden measurement and their characteristics.

**Materials and Methods:**

Based on Preferred Reporting Items for Systematic Reviews and Meta-Analysis (PRISMA) Extension for Scoping Reviews (ScR) guidelines, we conducted a scoping review assessing MEDLINE, Embase, Web of Science, and CINAHL from inception to April 2020 for studies investigating documentation burden among physicians and nurses in ambulatory or inpatient settings. Two reviewers evaluated each potentially relevant study for inclusion/exclusion criteria.

**Results:**

Of the 3482 articles retrieved, 35 studies met inclusion criteria. We identified 15 measurement characteristics, including 7 effort constructs: EHR usage and workload, clinical documentation/review, EHR work after hours and remotely, administrative tasks, cognitively cumbersome work, fragmentation of workflow, and patient interaction. We uncovered 4 time constructs: average time, proportion of time, timeliness of completion, activity rate, and 11 units of analysis. Only 45.0% of studies assessed the impact of EHRs on clinicians and/or patients and 40.0% mentioned clinician *burnout*.

**Discussion:**

Standard and validated measures of documentation burden are lacking. While time and effort were the core concepts measured, there appears to be no consensus on the best approach nor degree of rigor to study documentation burden.

**Conclusion:**

Further research is needed to reliably operationalize the concept of documentation burden, explore best practices for measurement, and standardize its use.

## INTRODUCTION

Rapid adoption of electronic health records (EHRs) following the passage of the Health Information Technology for Economic and Clinical Health (HITECH) Act has led to advances in both individual- and population-level health.^1^ HITECH has improved healthcare quality, patient safety, and diagnostic accuracy through enhanced data management and timely reuse; interoperable systems have facilitated care continuity and monitoring of compliance metrics.^2–5^ EHR-facilitated, guideline-based care has been associated with reduced redundancies^6^^,^^7^ and streamlined billing administration.^8^

Largely still in its infancy, the implementation of EHRs has also resulted in unintended consequences on clinical practice and healthcare systems, including significant increases in clinician documentation time.^9–13^ Extended work hours, time constraints, clerical workload, and disruptions to the patient-provider encounter, have led to a rise in discontent with existing documentation methods in EHR systems.^6^^,^^14^^,^^15^ This *documentation burden* has been linked to increases in medical errors,^3^^,^^9^^,^^16^ threats to patient safety,^3^^,^^9^^,^^16^ inferior documentation quality,^17^^,^^18^ job attrition, and, ultimately, burnout among nurses and physicians.^3^^,^^9–11^^,^^14^^,^^16–22^

In concert with Affordable Care Act (ACA) reimbursement models, Meaningful Use (MU) mandates, and a regulatory-rich environment, EHRs have drastically altered clinical documentation workflow and communication in routine healthcare.^13^^,^^15^^,^^23^ Physicians have reported willingness to remain out of compliance with EHR incentive programs (eg, MU and the Physician Quality Reporting System^24^) in favor of mitigating *documentation burden* (hereinafter referred interchangeably as “burden”).^15^^,^^25^ Still, studies consistently demonstrate that physicians spend twice as much time on electronic documentation and clerical tasks as compared to time providing direct patient care.^14^^,^^26–30^ Similarly, nurses devote more than half of their shift time to EHR data entry and retrieval^19^^,^^20^ and report reduced direct patient contact.^31^^,^^32^

While researchers have discussed the challenges of burden and its implications for clinician burnout due to EHRs over the past decade,^5^^,^^15^^,^^33^ limited attention has been paid to discriminating the antecedent concept of *burden* (defined as a duty, responsibility, etc, that causes worry, difficulty, or hard work),^34^ from *burnout* (defined as long-term work-related stress reaction marked by emotional exhaustion, depersonalization, and a lack of sense of personal accomplishment).^35^^,^^36^ Clinician burnout has been well-documented and widely quantified using surveys and psychological measurements throughout peer-reviewed literature.^37–40^ Yet, to our best knowledge, there is a lack of consensus on approaches to measure burden.^15^^,^^37^^,^^41–45^

While EHR dissatisfaction has been extensively studied and some clinician activity metrics have been proposed,^46^ few empirically-based readily-available solutions to reduce burden exist.^11^ Interventions to assuage burden have ranged from the utilization of scribes and remote transcription services ^27^ to text summarization and dictation software.^16^^,^^47^ In March 2020, the Department of Health and Human Services (HHS) released a report outlining 3 primary goals to reduce EHR-related clinician burdens that influence care: reduce the time and effort clinicians require to document health information, reduce the effort required to meet regulatory requirements, and improve EHR ease of use.^48^ Evaluating the impact of interventions that target these goals will necessitate standardized, quantitative measurements.

## OBJECTIVE

The purpose of this scoping review is to assess the state of science, identify gaps in knowledge, and synthesize characteristics of documentation burden measurement among physicians and nurses using EHRs.

## MATERIALS AND METHODS

We conducted a scoping review using the Preferred Reporting Items for Systematic Reviews and Meta-Analysis (PRISMA) extension for Scoping Reviews (ScR) guidelines.^49^ A scoping review fit our objective to describe the breadth of methods used to measure documentation burden.^49^

### Search strategy and selection criteria

We systematically searched the MEDLINE, Embase, Web of Science, and CINAHL databases for all English-language studies published in peer-reviewed journals and conference proceedings, investigating documentation burden among physicians and/or nurses in ambulatory and/or inpatient settings from inception to April 20, 2020. We evaluated all relevant literature identified through in-text references among eligible studies. Burden is not specifically represented in Medical Subject Headings (MeSH); therefore, we explored both keyword and MeSH terms for 2 burden-related concepts outlined in the HHS report *Strategy on Reducing Burden Relating to the Use of Health IT and EHRs*^48^ documentation: (a) effort, and (b) time. We also focused our search on: (a) the EHR and (b) physicians or nurses. The finalized search strategy is summarized in Table 1.

**Table 1.** Summary of search terms and query employed to each academic literature database in our review

Concept	Search Strings	Operator
documentation time	(“Task Performance and Analysis”[Mesh]) OR (“Costs and Cost Analysis”[Mesh]) OR (“Time Factors”[Mesh]) OR (“Process Assessment, Health Care”[Mesh]) OR (“time*”) OR (“Measure*”) OR (“measurement”) OR (“quantify”) OR (“quanti*”) OR (“metric”)	AND
documentation effort	(“Documentation*”[Mesh]) OR (“documentation*”) OR (“note*”) or (“unstructured data”) OR (“narrative”) OR (“Burnout, Professional”[Mesh]) OR (“Cognition*”[Mesh]) OR (“Cognitive load”) OR (“Burnout”) OR (“burden”)	AND
EHR	(“Electronic Health Records*”[Mesh]) OR (“electronic health record*”) OR (“electronic medical record*”) OR (“EHR”) OR (“EMR”) OR (“computerized medical record*”)	AND
physicians/nurses	(“Physicians”[Mesh]) OR (“Nurses”[Mesh]) OR (“nurse*”) OR (“physician*”)	

* Designates wildcard search.

### Study selection and selection criteria

We selected inclusion and exclusion parameters a priori, and iteratively modified them to exclude studies involving niche clinical systems and those strictly comparing to paper-based documentation (Table 2). We included all peer-reviewed primary studies that focused on EHR utilization with an objective time or effort measure^48^ (eg, EHR usage logs, which report time stamped documentation events) in the review.

**Table 2.** Inclusion and exclusion criteria

Inclusion	Exclusion
Peer-reviewed publications and conference proceedings Primary studies Objective time measures via EHR usage logs or other digital time capture tools Focus on EHR utilization Ambulatory setting and/or inpatient setting Physicians and/or nurses	Comparison to paper-based systems only Niche clinical systems (eg, radiology system, medication ordering system,) and applications Laboratory-based studies of prototype systems (ie, not yet used in clinical setting) Qualitative study Not accessible in the full text Not English language

The term “physicians” encompassed attending physicians, fellows, resident physicians, and interns; “nurses” referred to registered nurses. We focused on physicians and nurses given our aim of identifying interprofessional measurements of documentation burden. We excluded studies comparing EHR documentation to paper-based systems if they were not focused on measuring burden, but rather on EHR implementation evaluation.

After removing duplicates, 2 reviewers (AJM and JMS, AJM and RC, AJM and SS, or AJM and EL) independently screened article titles and abstracts for relevance using Covidence.^50^ Two authors (with a third serving as a tiebreaker) reviewed each potentially relevant abstract for eligibility criteria in the full-text. We included full-text articles with concordant decisions by the 2 reviewers in the final analysis; for discordant decisions, all reviewers reexamined and adjudicated until a consensus was reached.

### Data extraction and analysis

One author (AJM) performed data charting for all articles meeting full-text inclusion criteria (see online Supplementary Table), which was reviewed by all authors and discussed. We extracted the following information: publication year, geographic location, time source, unit(s) of analysis, activity, sample size, sample characteristics, EHR system, provider role/specialty, clinical setting, study design and objectives, study type (eg, quantitative or mixed-methods), site type (eg, single or multisite), exposure and outcome measures, analytical and statistical methods, study limitations/bias, and major findings. We reported study limitations and biases such as threats to internal and external validity to appraise rigor. We used the HHS concepts to organize our reporting of measurement characteristics.^48^ HHS does not elaborate further on definitions of: (a) time, (b) effort, and (c) outcomes assessed^48^; therefore, we conducted purposeful thematic analysis to identify proxies and synthesize these 3 recurring concepts.^51^ We iteratively combined themes until we achieved a consensus.

## RESULTS

### Sources of evidence

**Figure 1.**
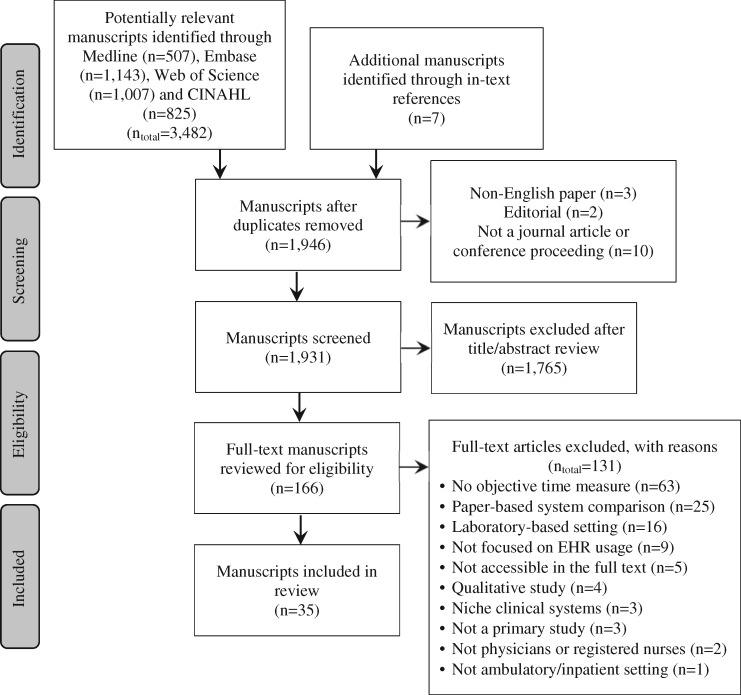
PRISMA flow diagram for scoping review of eligible studies.

Our search strategy yielded 3482 potentially relevant manuscripts from MEDLINE (n = 507), Embase (n = 1143), Web of Science (n = 1007), and CINAHL (n = 825). Seven additional manuscripts were identified through in-text references. After eliminating duplicates, 1946 titles/abstracts were screened; of those, 166 were eligible for full-text review. Consensus was achieved for all disagreements concerning the inclusion of full-text articles. Thirty-five studies meeting criteria were summarized in the final analysis (Figure 1).

### Study characteristics

Studies were conducted in the United States (n = 31),^13^^,^^14^^,^^22^^,^^27^^,^^28^^,^^43^^,^^52–76^ Europe (n = 1),^29^ and Asia (n = 3).^77–79^ Studies included a mix of ambulatory (n = 22)^14^^,^^22^^,^^27–29^^,^^52–54^^,^^56^^,^^61–66^^,^^69^^,^^71–75^ and hospital (ie, inpatient and emergency) settings (n = 11)^13^^,^^55^^,^^57^^,^^59^^,^^60^^,^^67^^,^^68^^,^^70^^,^^76^^,^^78^^,^^79^ with 2 involving both.^58^^,^^77^ A majority of those studies involved single sites (77.1%) and were affiliated with an academic institution/teaching hospital (80.0%). One third used Epic systems (n = 13),^13^^,^^22^^,^^27^^,^^43^^,^^53^^,^^54^^,^^56^^,^^57^^,^^64^^,^^66^^,^^69^^,^^72^^,^^73^ followed by multiple/other/unspecified (n = 12),^14^^,^^28^^,^^29^^,^^52^^,^^63^^,^^67^^,^^68^^,^^75–79^ Cerner (n = 6),^58–60^^,^^65^^,^^71^^,^^74^ Allscripts (n = 2),^61^^,^^62^ and Eclipsys (n = 2).^55^^,^^70^

Articles were published between 2010 and 2020 with 2018 (n = 8)^13^^,^^14^^,^^29^^,^^56^^,^^57^^,^^63^^,^^67^^,^^76^ and 2019 (n = 8)^22,54,58,69,72,74,6066^ representing the highest volumes. Range of study sample sizes was expansive among the studies (4 ≤ n ≤ 154 719). Most studies exclusively focused on physicians (n = 25)^13–2942^ as compared to nurses (n = 5)^58^^,^^67^^,^^76–78^ or an interprofessional sample of providers (n = 5).^22^^,^^55^^,^^56^^,^^69^^,^^73^ Clinician specialties were heterogeneous; over half the studies involved single specialties (general [n = 11],^14^^,^^27^^,^^52^^,^^53^^,^^61^^,^^62^^,^^64^^,^^71–73^^,^^75^ emergency [n = 2],^57^^,^^79^ intensivist [n = 2],^67^^,^^70^ other [(n = 5]^13^^,^^54^^,^^56^^,^^74^^,^^78^), while the remaining were multiple subspecialties (n = 13)^9^^,^^22^^,^^28^^,^^43^^,^^58–60^^,^^63^^,^^65^^,^^66^^,^^69^^,^^76^^,^^77^ or unspecified (n = 2).^55^^,^^68^ Across all studies, most involved general medicine (n = 17)^14^^,^^22^^,^^27^^,^^28^^,^^43^^,^^52^^,^^53^^,^^59–66^^,^^69^^,^^71–73^^,^^75–77^ followed by surgical subspecialties (n = 8),^13^^,^^29^^,^^58^^,^^59^^,^^66^^,^^74^^,^^77^^,^^78^ intensive care (n = 6),^58^^,^^59^^,^^67^^,^^70^^,^^76^ and emergency medicine (n = 4) ^57^^,^^58^^,^^60^^,^^79^; 10 included other subspecialties.^22^^,^^28^^,^^29^^,^^54^^,^^56^^,^^58^^,^^60^^,^^63^^,^^65^^,^^66^

Thirty were strictly quantitative studies. While purely qualitative studies were excluded, 5 studies employed mixed methods^28^^,^^52^^,^^55^^,^^61^^,^^62^ (see online Supplementary Table). Study designs varied, including time-and-motion (TM [n = 5]^28^^,^^61^^,^^62^^,^^67^^,^^68^), validation of TM (n = 2),^27^^,^^70^ cohort (n = 15),^13^^,^^27^^,^^43^^,^^54^^,^^55^^,^^57^^,^^59^^,^^60^^,^^63^^,^^64^^,^^66^^,^^72^^,^^74^^,^^75^^,^^77^ experimental/quasi-experimental (n = 8),^14^^,^^22^^,^^29^^,^^53^^,^^56^^,^^58^^,^^78^^,^^79^ and cross-sectional studies (n = 4).^69^^,^^71^^,^^73^^,^^76^ Eight studies evaluated an intervention,^14^^,^^22^^,^^52^^,^^53^^,^^56^^,^^58^^,^^75^^,^^78^ including scribes (n = 3),^14^^,^^52^^,^^53^ documentation redesign (n = 3),^58^^,^^75^^,^^78^ or EHR training programs (n = 2)^22^^,^^56^; the remaining were descriptive studies on EHR activities and usage (n = 27)—2 of which involved the implementation of new EHR systems.^29^^,^^79^

A diversity of analytical methods was employed. Most studies to which statistical testing were relevant (n = 23) applied parametric (n = 19) as opposed to non-parametric methods (n = 12). Qualitative methods employed in the mixed-methods studies involved informal interviews,^62^ social network analysis,^55^ thematic analysis,^62^ focus groups,^52^ and self-reported diary.^28^ Few studies addressed validity or reliability of measurements in their studies (n = 11) ^22^^,^^52^^,^^53^^,^^59^^,^^60^^,^^63^^,^^64^^,^^67^^,^^69^^,^^73^^,^^78^; 2 examined interobserver reliability,^28^^,^^68^ 2 employed TM approaches to validate novel analytical methods to examine workflow^70^ and the use of EHR usage logs to estimate workload,^27^ 2 examined correlations between self-reported and objective EHR usage log times,^22^^,^^73^ and 1 employed video recording timers to validate EHR usage log times.^58^

### Characterization of effort

Seven overarching *effort constructs* emerged (Table 3): (a) general workload such as overall EHR usage (n = 4)^53^^,^^56^^,^^68^^,^^69^; (b) clinical documentation/review (n = 15)^28^^,^^29^^,^^55^^,^^57–61^^,^^67^^,^^72^^,^^75–79^; (c) excess workload including EHR usage after hours (n = 15)^13^^,^^22^^,^^27^^,^^52–54^^,^^59^^,^^63–66^^,^^69^^,^^71^^,^^73^^,^^74^ and remote access (n = 1)^72^; (d) administrative tasks, such as inbox management (n = 2)^69^^,^^73^; (e) cognitively cumbersome work, such as multitasking (n = 3)^61^^,^^62^^,^^68^; (f) fragmentation of EHR workflow (n = 1)^70^; and (g) patient interaction/in-person visits (n = 7).^14^^,^^28^^,^^29^^,^^43^^,^^53^^,^^62^^,^^68^ Several terms were employed referring to EHR usage afterhours including “work after work,”^66^ “pajama time,”^66^ and “Clinician Logged-In Outside Clinic” (CLOC) time.^22^ For example, Cox et al proposed the “amount of EHR usage taking place after scheduled duty hours” specifically for surgical residents.^13^

**Table 3.** Identified measurement characteristics from study findings

Documentation Burden Concepts	Measurement Constructs	
Effort	EHR usage and workload	
	Clinical documentation/review	
	EHR work afterhours and remotely	
	Administrative tasks (eg, inbox management)	
	Cognitively cumbersome work (eg, multitasking)	
	Fragmentation of workflow	
	Patient interaction	
Time	Average time spent	
	Proportion or percentage of time spent	
	Binary of timeliness of completion (eg, documenting within shift or policy time frame)	
	Activity rate	
Units of analysis	Clinically-oriented units of analysis	Temporally-oriented units of analysis
	Encounter	Seconds
		Minutes
	Provider	Minutes
	Patient	Seconds
		Minutes
	Event/Task	Seconds
		Minutes
		Hours
		Shifts
		Days
		Weeks
		Months

*Note:* constructs and units are not intended to be comprehensive of all possibilities but rather reflect content identified in scoping review.

### Measurement of time

Time spent documenting was assessed in all studies and was measured using 3 key data collection strategies: EHR usage logs (n = 28),^13^^,^^14^^,^^22^^,^^27^^,^^43^^,^^53–60^^,^^63–67^^,^^69^^,^^71–79^ activity capture applications (n = 8),^27–29^^,^^52^^,^^61^^,^^62^^,^^68^^,^^80^ and video recordings (n = 1).^58^ Few studies triangulated these data through multiple data collection strategies (n = 2).^27^^,^^58^ Time constructs identified (Table 3) include (a) average time spent (n = 20),^22^^,^^27^^,^^29^^,^^43^^,^^54^^,^^55^^,^^57^^,^^59–61^^,^^63–67^^,^^69^^,^^71–73^^,^^78^ (b) proportion or percentage of time spent (n = 10),^13^^,^^28^^,^^53^^,^^56^^,^^62^^,^^68^^,^^70^^,^^72^^,^^74^^,^^75^ (c) binary of timeliness of completion (n = 1),^77^ and (d) activity rate (n = 2).^61^^,^^76^ Units of analysis varied within and across studies (Table 3), including time reported per: (a) encounter (n = 5),^54^^,^^60^^,^^65^^,^^67^^,^^69^ (b) provider (n = 2),^14^^,^^73^ (c) patient (n = 3),^57^^,^^59^^,^^78^ or (d) event/task (n = 28).^13^^,^^14^^,^^22^^,^^27–29^^,^^43^^,^^53–56^^,^^58^^,^^61–64^^,^^66^^,^^68^^,^^70–79^ Units of analysis also included average hours per day, per week, or per month (n = 6)^22^^,^^29^^,^^43^^,^^63^^,^^71^^,^^72^ and average minutes per day, per week, per shift, or per clinical full-time equivalent per week (n = 7).^27^^,^^55^^,^^61^^,^^64^^,^^66^^,^^73^^,^^78^ We have organized these units of analysis into 2 levels for combination in individual measures: (a) a clinically oriented unit of analysis, such as “per encounter,” and (b) a temporally oriented unit of analysis, such as “per hour” (see Table 3). Operationalization of a shift and “active versus idle” time in the EHR also varied. Among the 15 studies that examined shifts,^13^^,^^22^^,^^27^^,^^54^^,^^56^^,^^57^^,^^59^^,^^64^^,^^65^^,^^68^^,^^69^^,^^71^^,^^73^^,^^74^^,^^76^ 9 distinct shift times were identified with 6:00 am–6:00 pm (n = 4),^13^^,^^65^^,^^71^^,^^74^ 7:00 am–7:00 pm (n = 3),^69^^,^^73^^,^^76^ and 8:00 am–6:00 pm (n = 2)^22^^,^^27^ representing the most frequently reported intervals. Meanwhile, only half the studies employing EHR usage logs explicitly operationalized active versus idle time in the EHR to account for the time a clinician is logged in but not actively using the system. However, determination of “active and idle” time were measured at different levels of granularity (ie, complete system time-out [n = 3]^13^^,^^43^^,^^73^ vs “active versus idle” between tasks [n = 11]^22^^,^^27^^,^^56^^,^^59^^,^^60^^,^^64^^,^^65^^,^^69^^,^^71^^,^^72^^,^^74^). “Active versus idle” activity time was largely vendor defined (n = 7),^22^^,^^59^^,^^60^^,^^65^^,^^69^^,^^71^^,^^74^ relied on mouse clicks and keystrokes (n = 5),^59^^,^^60^^,^^65^^,^^71^^,^^74^ and/or idle time between 30 seconds and 10 minutes of length (n = 5).^27^^,^^56^^,^^64^^,^^69^^,^^72^

### Outcome assessment

Less than half the studies assessed the impact of documentation burden on clinicians and/or patients (n = 16). Among those studies, authors referenced the temporal relationship between burden and burnout at a higher proportion (68.8%) compared to those that did not extend beyond measuring time and effort alone (50.0%). Outcomes measured included clinical process measures [n = 8 (ie, treatment time, encounter closure, length of stay)^14^^,^^54^^,^^57^^,^^69^^,^^79^], clinician (n = 7)^14^^,^^22^^,^^52^^,^^53^^,^^75^^,^^78^^,^^79^ and patient satisfaction (n = 4),^14^^,^^52^^,^^53^^,^^63^ burnout/stress (n = 5),^22^^,^^64^^,^^69^^,^^73^^,^^75^ patient census/mortality (n = 2),^59^ response to messages (n = 1),^22^ and team interactions (n = 1).^55^ Primary predictors and outcomes of interest are summarized in the online Supplementary Table.

### Limitations and biases reported

Two limitations were ubiquitous across included studies (Table 4): (a) threats to *generalizability* due to constraints in sample size (n = 19),^14^^,^^28^^,^^29^^,^^52–54^^,^^57^^,^^59–64^^,^^67^^,^^69^^,^^70^^,^^73^^,^^74^^,^^79^ study setting (n = 21),^22^^,^^28^^,^^52–55^^,^^59–62^^,^^64^^,^^68–70^^,^^72–74^^,^^76–79^ patient population,^57^^,^^77^ EHR system (n = 6),^58^^,^^60^^,^^61^^,^^70^^,^^75^^,^^78^^,^^81^ activity type,^76^ clinician role or seniority,^57^^,^^59^^,^^61^^,^^69^^,^^70^ early adoption,^43^ and/or subspecialty;^62^^,^^64^^,^^70^ and, (b) *measurement error* including the inability of logs to distinguish between “idle and active” time (n = 6),^27^^,^^43^^,^^55^^,^^64^^,^^73^^,^^80^ uncertainty regarding the definition of “afterhours,”^59^^,^^73^ incomplete measurement of tasks (n = 15),^13^^,^^27^^,^^29^^,^^43^^,^^56–58^^,^^65^^,^^68–71^^,^^76^^,^^78^^,^^80^ imprecision of time capture,^27^^,^^43^^,^^55^^,^^73^^,^^80^ information bias (n = 10),^27–29^^,^^56^^,^^61^^,^^68^^,^^70^ and validity of measures.^53^^,^^54^^,^^64^

**Table 4.** Study limitations identified in the review

Author (Year)	generalizability	small sample size	selection bias	response bias	measurement error	misclassification	information bias	no data triangulation	confounding	self-reported data
Adler-Milstein et al (2020)	•	•			•	•				•
Ahn et al (2016)	•									
Anderson et al (2020)	•				•				•	
Arndt et al (2017)	•		•			•	•			
Aziz et al (2019)	•	•			•	•				•
Carlson et al (2015)	•		•	•	•				•	•
Collins et al (2018)	•				•			•		
Cox et al (2018)	•				•	•		•	•	
DiAngi et al (2019)	•		•	•	•					•
Earls et al (2017)	•	•	•		•		•	•	•	•
Gidwani et al (2017)	•	•			•			•		•
Goldstein et al (2019)	•	•			•			•	•	
Hripcsak et al (2011)	•				•			•		
Hsieh et al (2016)	•				•					•
Inokuchi et al (2015)	•	•	•	•						•
Joukes et al (2018)	•	•	•		•		•		•	
Kadish et al (2018)	•			•	•		•	•		•
Kannampallil et al (2018)	•	•	•		•	•			•	
Karp et al (2019)	•				•					
Krawiec et al (2019)	•	•			•			•		
Krawiec et al (2020)	•	•			•				•	
Mamykina et al (2012)	•	•			•		•			
Mamykina et al (2016)	•	•	•		•		•		•	
Marmor et al (2018)	•	•			•			•	•	•
Micek et al (2020)	•	•		•	•	•				•
Mishra et al (2018)		•	•				•	•		•
Overhage et al (2020)	•				•	•			•	
Saag et al (2019)								•		
Sinsky et al (2016)	•	•	•		•		•		•	•
Smith et al (2018)	•	•			•					
Tai-Seale et al (2017)	•		•		•	•				
Tipping et al (2010)	•				•		•			
Tran et al (2019)	•	•			•				•	•
Wang et al (2019)	•				•					
Zheng et al (2010)	•	•	•		•		•			

Six studies cited selection bias derived from both the presence of self-selection and voluntary participation among high-performing subjects^27^^,^^28^ and the presence of low response.^22^^,^^56^^,^^64^^,^^75^ Eleven studies noted a lack of data triangulation, such as combining log data with direct observations, encounter information or qualitative data to offer contextual information corresponding to types of EHR interfaces used (eg, remote, inpatient, outpatient) for login timestamps, direct patient care, and other data.^13^^,^^14^^,^^52–56^^,^^60^^,^^63^^,^^66^^,^^76^ Twelve studies identified the presence of potential confounding.^13^^,^^28^^,^^29^^,^^52^^,^^54^^,^^57^^,^^59^^,^^62^^,^^63^^,^^65^^,^^69^^,^^71^^,^^75^

## DISCUSSION

In this scoping review, we identified 35 studies that explored the measurement of documentation burden among physicians and nurses, underlining the overall paucity of research in the domain. As may be expected, all 35 studies were published post-HITECH Act. Seven effort constructs, 4 time constructs, and 11 units of analysis were uncovered. Our effort constructs—except *workflow fragmentation* and *cognitively cumbersome work* (eg, multitasking)—largely align with “proposed core EHR use measures (for practice efficiency)” published by Sinsky and colleagues which indicates burden may be quantified through existing metrics.^46^ Generated with expert stakeholders, Sinsky’s core measures include total EHR time, work outside of work, time on documentation, time on prescriptions, inbox time, teamwork for orders, and undivided attention to patients.^46^ Further efforts should examine these measures for validity and reliability. Fewer than half (n = 16) of the studies investigated the impact of burden on clinicians and/or patients. Methodologies varied across study design, suggesting there is no current consensus regarding best approach or standard to study burden, although it is possible an ensemble of methods coupled with the triangulation of multiple data sources will emerge as a best practice.

Historically, TM studies have been considered the gold standard for quantifying the effects of computer systems on task-based clinical workflow and duration.^82^^,^^83^ Despite yielding valid results,^70^^,^^84^^,^^85^ TM studies are costly and time-consuming to perform^83^ and engage only a handful of participants per study. In addition to concerns regarding the generalizability of TM studies, prior research has identified widespread methodological inconsistencies in their design and conduct as well as in their quantitative analyses and reporting of results, making it difficult to synthesize findings across studies.^70^^,^^86^ Readily accessible and scalable, and less subject to the Hawthorne effect, evidence may suggest that analyzing EHR usage logs is a more feasible alternative as these data were used in the overwhelming majority of included studies (80.0%). Nevertheless, research on the use of EHR usage logs to evaluate clinical activity has revealed a dearth of validation, cross-study analyses, and, most critically, defined terminology (eg, access log, audit log) and measures.^46^^,^^87^ These inconsistencies parallel those found in TM studies, as described above. TM studies provide valuable contextual information on time and sequence of activities performed which can be triangulated with EHR usage logs to better understand burden in the context of clinical workflows. In recognizing that all methods have strengths and weaknesses, we anticipate that future work will identify the methods of measurement and triangulation of data that best align with different research objectives related to burden.

One major finding of this review was the absence of quantitative studies assessing the reliability and validity of time and effort measures. Of the 35 studies included, only 1 study intended to develop a measure of burden (ie, EHR usage outside shift),^13^ while 2 studies individually employed TM studies to empirically validate proposed measures of workflow and the use of EHR usage log data in characterizing workload.^27^^,^^70^ Interobserver reliability was reported in only 2 studies.^28^^,^^68^ As described above, previous studies on quantifying physician EHR activity through EHR usage logs have noted similar challenges.^87^ The lack of studies developing and validating burden measures confirms that limited efforts have been dedicated to formally and objectively quantifying and measuring burden, despite increasing references to it in public policy and lay literature. Researchers have often used unstandardized proxies to quantify burden which elucidates why no objective proxies exist.^6^^,^^13^^,^^14^^,^^25^ Reinforcing the absence of empirical validation studies, there is a lack of an agreed-upon definition for *burden* and a plethora of definitions throughout the literature.^6^^,^^13^^,^^14^^,^^25^^,^^28^^,^^43^^,^^47^^,^^52^^,^^88–90^ We found that many related—but different—concepts were used in the context of studies quantifying *time* and *effort,* such as workload,^27^^,^^78^ workflow,^13^^,^^74^ work disruption,^75^ efficiency,^22^^,^^52^ cognitive burden,^56^ usability,^74^ and productivity, among others.^69^ In contrast, *burnout* is identifiable in controlled vocabularies including, the International Classification of Diseases (ICD), in addition to the Diagnostic and Statistical Manual for Mental Disorders (DSM) and MeSH.^91^^,^^92^ Furthermore, validated measures of burnout, such as the Maslach Burnout Inventory and the Mini Z burnout survey are often applied,^69^^,^^73^ whereas no known analog for burden is currently available. Likewise, in a literature review conducted on the impact of EHRs on documentation time among physicians and nurses, Poissant and colleagues suggested that a lack of research evaluating EHR time efficiency is likely associated with the poverty of rigorous methods accurately capturing *time.*^12^ We found that generalizability and measurement error issues were partially driven by the use of distinct EHR systems with some instances of proprietary and opaque vendor-defined time metrics for shift and active EHR time.^73^ There was also imprecision in time capture among EHR usage log studies. Reported elsewhere in the literature, EHR usage logs have exhibited unreliable degrees of accuracy for both clinician activity and time durations captured.^87^ Intended for troubleshooting technical problems and HIPAA compliance, EHR usage logs originate from many interconnected information systems and sources (eg, devices).^93^ Vendor-defined time metrics may not be generalizable between, or within, institutions or provide precise estimates in real-world settings. Therefore, given the value in measuring clinician EHR time, researchers should explore novel algorithmic methods to validate these metrics and EHR usage log data. For example, Dziorny and colleagues developed an automated algorithm to quantify shift duration among physicians in an inpatient setting and internally validated it against scheduled shift-time.^83^ Likewise, DiAngi et al proposed the “calculated EHR time outside of clinic” (CLOC) metric for ambulatory settings to measure after clinic hours using EHR usage logs and were able to correlate their findings with self-reported time spent in the EHR after clinic hours.^22^

The HHS Report—*Strategy on Reducing Burden Relating to the Use of Health IT and EHRs*—aims to evaluate the clinical impact of burden (ie, *time* and *effort*) on clinicians and/or patients;^48^ however, fewer than half the studies reviewed investigated an outcome of interest (n = 16). Of those studies (note: outcomes were not mutually exclusive), the majority examined clinician satisfaction and burnout (n = 12), while only half examined clinical process measures as an end goal. Half evaluated patient satisfaction and health indicators. Research questions and study objectives were widespread across included studies.

In this review, scribes represented 1 of 3 areas of study concerning proposed interventions to mitigate burden (n = 3);^14^^,^^52^^,^^53^ however, associated costs and high turnover rates among scribes suggest that this solution may not be broadly feasible or sustainable.^47^ In the context of reducing documentation burden, implementing and measuring the impact of scribes does not solve the higher-level information processing issues that informatics research should be investigating (eg, reduction in data entry requirements, improvement of system usability) and possibly diverts resources away from more sophisticated biomedical informatics approaches. Other identified interventions, such as training on EHR use (n = 2)^22^^,^^56^ and documentation redesign (n = 3)^58^^,^^75^^,^^78^ also have their strengths and weaknesses. Training may represent a lower cost method of mitigating burden than scribes, while documentation redesign may be more costly but likely more effective at solving information processing and usability concerns. Moreover, lack of standardized measures leads to the inability to conduct comparative effectiveness studies on design modifications within EHR systems^15^ or across distinct burden-alleviating interventions.

In summary, our findings identified distinct, but not necessarily comprehensive, characteristics of measuring burden: 7 *effort constructs,* 4 *time constructs*, and 11 units of analysis (see Table 3).

### Limitations

While this study sought to investigate literature on the operationalization of documentation burden and the development and/or validation of quantitative burden measures, research in this domain has not yet matured. Despite employing broad search terms and queries, the majority of the literature retrieved did not detail how to conceptualize and/or measure burden. We extracted manuscripts using keywords, as extant MeSH terms were unable to capture the phenomenon of study interest; in fact, no term for *burden* used in this specific context exists. It is conceivable that some articles were not captured because: (a) our keywords were limited, and/or (b) our queries were not sufficiently broad or narrow.

### Future directions

Future research should build upon existing burden evidence, focusing on strengthening objectivity and generalizability. Proposed quantitative measures of burden such as the *after scheduled duty hours* measure described by Cox and colleagues should undergo rigorous testing and validation across settings and specialties.^13^ Additionally, HHS links time and effort concepts to clinical impact; ^48^ therefore, research should directly connect measurement of these concepts with specific outcome measures to be able to accurately evaluate documentation burden over time. This remains a difficult undertaking as studies have shown that neither burden nor task value in the clinical context are identical across all EHR interactions or across different roles and specialties.^10^^,^^20^^,^^25^ Examining tradeoffs between specific tasks within the EHR, Rao and colleagues discovered that EHR functionalities are not equally burdensome.^25^ They also found that settings are not equally burdensome, reporting that shift-based work may be associated with less burden and that ambulatory clinical documentation is rated equally valuable and burdensome.^25^ Perceptions of distinct documentation types among nurses have also been studied, yet no objective criteria have been established to evaluate value.^19^ We found that only 1 study investigating EHR work afterhours (ie, “pajama time”) included nurses.^77^ While “pajama time” connotes remotely accessing the EHR from home to document, few inpatient nurses do so given the immediacy of their documentation. Thus, data entry rates may be more suitable for measuring nurse burden.^76^ Because physicians working in general medicine were most represented in our findings, future work should be dedicated to characterizing and measuring burden among understudied professions and settings (ie, nurses and subspecialties).

However, promisingly, burden measures identified were not strictly unique to individual professions and workflows, supporting the opportunity for defining interprofessional measures of burden in future work. We propose that burden be examined as a global composite measure, indicative of magnitude and directionality, consistent with the characteristics uncovered in this review. This would require: (a) developing a universally agreed-upon inventory for key EHR tasks and activities weighted for relative value according to burden (ie, a taxonomy) that could be linked to clinical outcomes such as “quality, financial or professional satisfaction”^15^^,^^27^^,^^28^; and (b) quantifying the relationship between “pain points” and specific features in the EHR with more granularity. This furnishes the examination of *task value*, as indicated by task relationship with burden, a high priority area for future research. Such research would allow the identification of tasks that are of high burden but low value so that EHR design and intervention efforts may target the elimination or mitigation of these tasks.

## CONCLUSION

Documentation burden among interprofessional clinical roles remains understudied and under-measured in both inpatient and ambulatory settings. This review suggests that concrete, validated measures of burden in research are lacking, which pales in comparison to burnout literature.^36^ Moreover, this review demonstrates that the existing evidence is imprecise and fragmentary. While there is a multitude of measures for both effort and time among the included studies, the majority lack generalizability across study setting, patient population, EHR system, activity type, role, and subspecialty. In the absence of standardization, these studies additionally run the risk of measurement error including misclassification of idle and active time, completeness of task measurement, and precision of time capture. Hence, it would be prudent to further explore easily accessible, scalable alternatives, such as EHR usage log data. Targeting burden to evaluate the impact of quality improvement strategies and interventions requires quantifiable measures that are comparable and consistent across time, settings, professions, and contexts. We propose that burden should be examined as a global composite measure based on task value, consistent with burden measurement characteristics uncovered in this review. Further research is needed to reliably operationalize and standardize the concept of burden and to explore how it is best measured across clinical settings.

## FUNDING

This study was supported by the US National Library of Medicine of the National Institutes of Health (NIH) under the training fellowship award 5T15LM007079 and the National Institute for Nursing Research (NINR) under grant numbers 1R01NR016941 and 5T32NR007969.

## AUTHOR CONTRIBUTIONS

AJM and SCR conceptualized the scope of this review. AJM, JMS, RC, SS, and EL conducted the initial and full-text screenings. AJM drafted the manuscript with significant revisions and feedback from JMS, RC, KDC, and SCR.

## SUPPLEMENTARY MATERIAL

Supplementary material is available at *Journal of the American Medical Informatics Association* online.

## DATA AVAILABILITY STATEMENT

The data underlying this article are available in the article and in its online supplementary material.

## CONFLICT OF INTEREST STATEMENT

None declared.

## Supplementary Material

ocaa325_Supplementary_Data
