# Supplementary material for: Measurement of clinical documentation burden among physicians and nurses using electronic health records: a scoping review
Source: J Am Med Inform Assoc. 2021 Jan 12;28(5):998–1008. doi: 10.1093/jamia/ocaa325 (PMC8068426; doi:10.1093/jamia/ocaa325)
Supplement: ocaa325_Supplementary_Data [file ocaa325_supplementary_data.docx]

| **Author (Year); Country** | **Time Source(s)** | **Effort; Unit(s)** | **EHR System** | **Population (n); Setting; Specialty** | **Study Design (Analytical Methods)** | **Main Predictor(s); Outcome(s)** | **Measure(s)** | **Major Findings** | **Study Limitations Identified** |
| --- | --- | --- | --- | --- | --- | --- | --- | --- | --- |
| Adler-Milstein et al. (2020); USA | EHR log, Provider Efficiency Profile measures | *effort*: time after hours on clinic days, time on nonclinic days, message volume, efficiency and proficiency; *unit(s):* minutes per clinical full-time equivalent per week | Epic Systems | physicians and nurse practitioners (n=87); ambulatory; primary care | cross-sectional study (summary statistics, correlations, bivariable analysis, logistic regression, multivariate regression) | *predictor(s)*: EHR use; *outcome(s)*: cynicism and exhaustion | *work experience survey:* 5-item emotional exhaustion subscale, 5-item cynicism subscale from Maslach Burnout Inventory General survey, 5-point Likert-type scale for EHR proficiency, 5-point Likert-type scale for time spent on EHR at home; *EPIC Provider Efficiency Profile (PEP) measures*: time active after hours (7:00pm-7:00am) on scheduled clinic days, time active anytime on unscheduled days, volume of received messages (per week per 1.0 clinical FTE), frequency of use of available EHR tools (proficiency), time clinicians spend in EHR relative to expected time based on clinical workload (efficiency); *administrative data*: patient panel size, panel complexity, FTE effort allocated | Perceived burden of EHR time spent at home correlated with PEP EHR workload measures; EHR time after hours on clinic days and message volume associated with greater odds of high exhaustion among clinicians; PEP measures were not associated with cynicism; no association between proficiency and efficiency, and composite measures with cynicism or exhaustion. | generalizability, self-reported data, compositive PEP measures proprietary to EPIC (e.g., EHR logs cannot differentiate idle time vs. active time, opaque definition of afterhours), small sample size, subjective EHR measures |
| Ahn et al. (2016); South Korea | EMR timestamp  Data | *effort:* documentation outside of working hours; *unit(s)*: count, proportion | not specified | nurses (n=99); ambulatory and inpatient; internal medicine, surgery | retrospective study (descriptive statistics, logistic regression) | *predictor(s)*: nurse- and patient-associated factors; *outcome(s)*: timeliness | *timeliness (timely/untimely)*: electronic documents entered between within working hours of shift (6:00am–2:00pm for day shift, 2:00pm–10:00pm for evening shift, 10:00pm–6:00am for night shift); *nurse- and patient-associated factors* | Significant increased odds of timely documentation among experienced nurses compared to less experienced nurses, and evening and weekend shifts compared to weekday shifts; surgery department nursing documentation significantly more timely than internal medicine documentation. | generalizability (clinical environments, units, and patient populations) |
| Anderson et al. (2020); USA | EHR usage data (Cerner Advance) | effort: time spent after hours; *unit(s)*: average hours per month | Cerner | physicians (n=34); ambulatory; family medicine | cross-sectional study (descriptive statistics) | *predictor(s)*: n/a; *outcome(s)*: number of patients seen, total time per patient and total time spent afterhours | *Cerner Advance EHR usage data, EHR afterhours use*: time spent in EHR between 6:00pm-6:00am and on weekends; number of patients seen and total time spent per patient | Physicians spent a large amount of time completing EHR tasks, including a large proportion after hours; time ranges across physicians varied independent of training. | generalizability, faculty EHR time difficult to measure, confounding, data collected around EHR transition period, vendor-defined active time |
| Arndt et al. (2017); USA | event log data, WorkStudy+ application | *effort*: time spent after hours, time spent on inbox management; *unit(s)*: minutes per day | Epic Systems | physicians [n=14 (time and motion validation study], n=142 (log files)]; ambulatory; family medicine | retrospective cohort study, time-and-motion study (descriptive statistics) | *predictor(s)*: n/a; *outcome(s)*: time spent in the EHR | *EHR event logging data*: average total EHR time per weekday per 1.0 clinical FTE; EHR hours after clinic on weekends and weekdays (total EHR time after 6:00pm on Fridays through 8:00am on Mondays divided by 5 days) | PCPs spent more than half their hours worked during and after clinic interacting with computer; two-thirds of computer time was spent on clerical and inbox tasks. | generalizability, volunteers, subroutines and tasks within an encounter (e.g., telephone call) not evaluated, event logs cannot determine between active time spent in EHR versus open EHR engaging in other activities, only measures EHR time not total patient care time, does not account for volume of faxes and other paperwork, possible underestimation of other work outside of clinic hours, observation data |
| Aziz et al. (2019); USA | timestamp data (Cerner Advance), self-reported duty hours | *effort*: time spent in EHR after hours, time spent per patient chart after hours, time spent per patient chart; *unit(s)*: proportion of total EHR time (hours per week) | Cerner | physicians (n=11,812 charts); ambulatory; vascular surgery (surgical specialty) | retrospective observational study, pilot study (descriptive statistics) | *predictor(s)*: on and off duty hours; *outcome(s)*: EHR-related activities usage time | *Cerner Advance EHR activity*: overall EHR activity time, chart review time (time spent reviewing clinical documents, patient flowsheet, and medication administration record), electronic order entry time, documentation time, patient discovery time (time spent on activities not mentioned), and electronic messaging time; *time of use*: number of on-duty hours (6:00am-6:00pm), number of off-duty hours (6:00pm-6:00am); *self-reported activity*: duty start time, duty end time; *time spent per patient chart*: total amount of time spent on EHR over total number of patient charts accessed | Participants spent one fifth of time spent in the EHR after hours, of which chart review accounted for the most time followed by order entry and documentation; on average, time spent per patient chart after hours higher than time spent per patient chart during duty hours. | generalizability (single institution), small sample size, retrospective observational study, vender-defined active time, self-reported data |
| Carlson et al. (2015); USA | EHR timestamp data | *effort*: time spent on documentation; *unit(s)*: median percentage of days | locally-developed EHR; StarPanel | physicians (n=91); ambulatory; primary care | longitudinal observational study, pre- and post-intervention survey (Fisher's Exact test) | *predictor(s)*: structured data-entry forms; *outcome(s)*: timeliness rate of completion | *timeliness*: days to completion of resident documentation; days to completion of attending attestations; *satisfaction survey*: demographics, time spent on documentation, perceived accuracy of physical exam/list of diagnoses, usefulness of current documentation form, and documentation satisfaction with and stress | Median percentage of timely documentation or attestations increased for residents and attendings post-implementation; significant increase in residents and attendings reporting high satisfaction with documentation; significant decrease in residents reporting high stress with documentation. | convenience sampling, generalizability (EHR), increased timeliness perhaps due to new incoming residents, low post-implementation response rate (bias), possible regression towards the mean, self-reported data |
| Collins et al. (2018); USA | EHR timestamp data | *effort*: data points per 12-Hour shift; *unit(s)*: data points per hour (mean) | not specified | nurses (n/a); inpatient; acute care general medicine (GMU) and medical intensive care units (MICU) | cross-sectional study (descriptive statistics) | *predictor(s)*: n/a; *outcome(s)*: documentation burden (flowsheet data entry rate) | *flowsheet data*: mean and standard deviation of flowsheet data entries per nurse per 12-hour day shift (7:00am–6:59pm) or 12-hour night shift (7:00pm–6:59am), mean and standard deviation of users documenting, mean data points per user; *shift*: last data entry minus first data entry; *device integrated data elements* | Flowsheet data entry rates appeared similar between ICUs and acute care units; differences found among acute care units during night shift; rates during the night shifts higher for each unit than corresponding rates during the day; greater portion of device integrated data captured in the ICU than in acute care units; accounting for device integrated data points, nurses in ICU and acute care units exhibited similar flowsheet data entry rates. | generalizability (single institution, one type of one type of EHR documentation, focused on EHR data entry only), EHR log-file data without direct observation, does not evaluate temporal data entry trends, possibly multiple data enterers in flowsheet documentation |
| Cox et al. (2018); USA | EHR timestamp data | *effort*: EHR usage after hours; *unit(s)*: proportion of total EHR usage (per week) | Epic Systems | physicians (n=36); inpatient; general surgery | retrospective cohort study (Fisher's Exact test, Wilcoxon rank-sum test, longitudinal linear mixed-effects models, Pearson’s correlation coefficient, sensitivity analysis) | *predictor(s)*: n/a; *outcome(s)*: amount of time logged into the EHR system per resident stratified by day of week and time of day | *active EHR use (for concurrent logins)*: timeframe between login and next consecutive logout; *logout timestamp*: system (20 minutes or 40 minutes of inactivity or logout of desktop) or user logout; *proportion of EHR usage during and after hours* (daytime hours: 6:00am-5:59pm, nighttime hours: 6:00pm-5:59am), EHR usage time during the designated 12-hour shift divided by the total time of EHR usage; *other study data*: demographics, monthly operative case logs, rotation schedules | Residents spent significantly more time logged into the EHR during week compared to weekends and during day compared to nights; night rotation residents spent 7% of login time on average outside regularly scheduled duty hours; day rotation residents spent 27%; residents spent one third of EHR usage outside the scheduled 12-hour shift; no correlation between operative cases logged monthly and EHR usage. | unmeasured confounders, granularity, EHR usage determined by login/logout timestamps but work type undetermined, unknown interface type used for each login timestamp (i.e., inpatient, outpatient, or remote), possible underestimation of EHR use, generalizability |
| DiAngi et al. (2019); USA | timestamped log | *effort*: time spent working in the EHR during personal time, Clinician Logged-In Outside Clinic Time (CLOC Time); *unit(s)*: mean hours per week | Epic Systems | providers (n=561); ambulatory; primary care, obstetric, behavioral health and subspecialty providers | descriptive exploratory operational study, pre-post study with no controls (descriptive statistics, paired t-tests, ANOVA, Pearson correlation) | *predictor(s)*: supplemental EHR training program; *outcome(s)*: self-reported data, calculated EHR time and vendor-reported metrics | *EHR/perceived experience variables* (Mini-Z burnout survey): EHR satisfaction, clinical work, face-to-face time with patients, EHR workload, satisfaction with time spent in EHR after clinic hours, EHR competence, stress level related to the EHR, and self-reported time spent in the EHR after clinic (hours); *EHR functionality questions* (Smart-Phrases, preference lists, filters, closing visit notes): seven items on frequency of use of EHR functions, seven items on knowledge of common EHR functions, five items on ease of use of EHR functions; *vendor-reported metrics (EHR-use metrics)*: provider response time, inbox turnaround time, preference list entries; *Clinician Logged-In Outside Clinic Time (CLOC Time)*: time logged into EHR after clinic hours (i.e., evening, weekend, and vacations) with standard clinic hours defined as a half hour before the start of the first scheduled appointment through the end time of the day’s last visit plus 1 h); ratio of hours using the EHR outside of scheduled patient care time to the number of hours a provider is open/scheduled to see patients | Self-reported time and CLOC time highly positively correlated, but ratio of self-reported time versus measured CLOC time was 2:3; significant increase in self-reported knowledge of efficiency tools in EHR and in preference list entries after training; only self-reported control over workload significantly improved after training; no significant decrease in calculated EHR usage outside of clinic after training, or difference in turnaround time for patient calls and results; significant differences seen in self-reported EHR hours and CLOC time for post-intervention satisfaction with workload and satisfaction with time spent in the EHR after clinic hours. | multiple comparisons (p-value adjustment), low completion rate, non-random enrollment (response bias), single academic pediatric institution, no control group, CLOC not generalizable or normalized, difference in provider self-reporting of after clinic hours vs. CLOC time (i.e., provider overreporting or algorithm undermeasuring) |
| Earls et al. (2017); USA | smartphone time-tracking application | *effort*: time spent at home working on clinical documentation; *unit(s)*: hours per week | not specified | physicians (n=6); ambulatory; family medicine | pilot mixed-method quality improvement study, retrospective review, focus groups (univariate statistics, bivariate statistics, nonparametric comparisons) | *predictor(s)*: scribes; *outcome(s)*: physician work-life balance and provider satisfaction | *time-tracking application*: hours spent at BFHC and at home working on clinical documentation; *EMR variables*: total clinical hours, number of clinic sessions (consecutive uninterrupted appointments), average hours per session, number of patient appointments scheduled per session, and number of patient visits conducted per session; *physician work-life balance*: 19 items from Physician Work-Life Survey (5-point Likert scale); *patient satisfaction*: six closed-end questions on comfort level with the scribe in exam room, willingness to have a scribe present for subsequent visits, importance of scribe being same gender/age as patient, overall satisfaction with scribe presence; provider satisfaction: five closed-end questions on comfort level with scribe presence, ease of EMR documentation, change in office hours with scribe for day’s session(s), overall scribe helpfulness; *impact assessment*: patient volume, patient satisfaction, visit flow, EMR documentation. | Physicians reported high satisfaction with scribes with better ability to remain on schedule, assistance in finding important record information, and having notes completed at end of sessions; decrease in time spent in clinic and time spent working at home post-intervention despite increase in average scheduled clinic hours over study period. | small sample size, generalizability (single institution), information bias in patient/provider feedback (social desirability), possible bias due to absence of rigorous qualitative research, no comparison group, clustering by physician and/or scribe not possible, self-reported data |
| Gidwani et al. (2017); USA | EHR timestamp data | *effort*: time to chart close; *unit(s)*: proportion | Epic Systems | physicians (n=4); ambulatory; family medicine | randomized controlled trial (descriptive statistics, fixed-effects logistic regression with Bonferroni correction, fixed-effects logistic regression adjusted for clustering, sensitivity analyses) | *predictor(s)*: scribes; *outcome(s)*: physician satisfaction, patient satisfaction and charting efficiency | *physician satisfaction*: self-administered 5-item questionnaire (7-point Likert scale); *patient satisfaction*: shortened, validated, 6-item questionnaire designed for the primary care setting (7-point Likert scale); *physician efficiency*: time to chart close (time from appointment start to physician signing chart note). | Scribes significantly associated with physician satisfaction in all measured domains of patient care and documentation (e.g., satisfaction with their clinic day, face time with patients, amount of time spent charting); significant increase in physician satisfaction with quality and accuracy of their charts when scribe was present; scribed charts significantly more likely to be closed within 48 hours compared to physician only charts. | generalizability (small sample size, single clinic), randomization at physician-week level, physician satisfaction instrument not validated, less objective than time and motion study, evaluation of chart quality not objective (unblinded observers) and instrument not validated, self-reported data |
| Goldstein et al. (2019); USA | audit logs | *effort*: time spent using EHR, chart closure time, note length; *unit(s)*: hours per task, average minutes per office visit | Epic Systems | physicians (n=70); ambulatory; ophthalmology | longitudinal cohort study (descriptive statistics, linear mixed models, Tukey test with Holm-Bonferroni method, type II Wald chi-squared test) | *predictor(s)*: Meaningful Use period/years; *outcome(s)*: time spent using EHR, chart closure time and note length | *EHR time spent per office visit*: number of distinct minutes in provider’s relevant audit log entries for each visit (multiple office visits were accessed in the same minute proportionally divided); *note length*: sum of characters in all office visit progress notes; *chart closure time*: check-in time subtracted from close date time | Significant increase in note length over time which was independent of MU period; EHR time per office visit from 2014 through 2016 was significantly longer compared to 2006 through 2010; chart closure time from 2014 through 2016 was significantly quicker than 2006 through 2010; trends in EHR time spent per office visit and chart closure varied with the adoption of federal MU guidelines. | generalizability (single academic institution and EHR), retrospective study, cannot establish causation, individual compliance with MU not evaluated, validated measure for EHR time per office may be outdated, no note text stored in data warehouse, only office visit audit log data evaluated, possible confounding on scribe or trainee presence |
| Hripcsak et al. (2011); USA | audit logs | *effort*: time spent authoring, time spent viewing; *unit(s)*: minutes spent per day and week (mean) | Eclipsys XA | 19 user groups including nurses, physicians, and attendings (n=4,121); inpatient; n/a | longitudinal observational study, social network analysis (descriptive statistics, social network analysis) | *predictor(s)*: n/a; *outcome(s)*: time spent authoring, time spent viewing and network relationships | *authoring*: mean rate per week, mean time per day; *viewing clinical documentation*: mean rate per week, mean time per day; *associations among users*: degree of the network (mean number of viewings by a viewer of an author’s notes), assortative mixing among user groups (pairwise associations among user groups), hierarchical community structure (larger groupings of users viewing each other’s notes) | Most user groups spent less than 90 minutes per day authoring and viewing notes; overall 16% of notes went unread; proportion that went unread occurred varying degrees across user groups; notes were accessed after 2 years, but note viewing decreased with note age; nearly all healthcare teams comprised of a nurse, attending, and resident, who also were the user groups who wrote the first note at admission. | generalizability (single academic medical center), use of log files without direct observation, oral communication not captured, does not account for system idle time, underestimation of nursing documentation time (excludes flowsheets) |
| Hsieh et al. (2016); Taiwan | computer registry log | *effort*: time on documentation; *unit(s)*: time per patient per day (minutes), time per shift per day (minutes) | not specified | nurses [n=20 (pre-implementation), n=19 (post-implementation)]; inpatient; surgical unit | quality improvement project, pre-and-post study (descriptive statistics, Wilcoxon two-sample test, Wilcoxon rank-sum test) | *predictor(s)*: redesigned evidence-based focus templates; *outcome(s)*: documentation time and satisfaction | *documentation time*: difference between computer registry log start time and end time for documenting each focus; *self-administered focus questionnaire (nurse satisfaction)*: satisfaction with content, functionality, effectiveness, and usability of new focus database (5-item Likert scale) | Total documentation time per week decreased comparing pre-implementation to post-implementation; median documentation time per patient per day decrease by nearly a half; no significant change in documentation time for night shift; satisfaction with usability significantly increased post-implementation. | generalizability (single surgical unit with longer documentation time than other surgical units in institution, electronic charting system not widely used in Taiwan), focus questionnaire internally developed and not validated, no overtime tracking system, self-reported data |
| Inokuchi et al. (2015); Japan | EMR timestamp data | *effort*: time entering patient clinical data; *unit(s)*: mean timespent (minutes) | IBM | physicians (n=6), patients (n=526); emergency department; emergency medicine | randomized crossover design (independent t-tests, chi-squared tests) | *predictor(s)*: EMR implementation; *outcome(s)*: medical treatment time, length of stay and physician satisfaction | *time for overall medical care* (time between start of medical treatment to final clinical documentation entry); *physician satisfaction:* 5-point Likert scale*; rate of admission*: patient flow, overcrowding, length of stay in ED; *patient demographics*; *patient acuity*: Japanese Triage and Acuity Scale | Post-implementation significant decrease in mean time spent on clinical documentation; significant reduction in time spent on overall medical care; physicians reported high EMR satisfaction in ability to capture images and enter negative findings, and the physical findings support system. | generalizability (single center), small sample size, short observation period, participant bias towards EMR, self-reported data, patient outcomes not assessed |
| Joukes et al. (2018); Netherlands | online application | *effort*: documentation time, time for patient care; *unit(s)*: percentage time spent | not specified | physicians (n=24); ambulatory; pediatrics, gynecology/obstetrics, urology, endocrinology, pulmonology, traumatology, orthopedic surgery, hematology, ophthalmology, nephrology, otorhinolaryngology, neurology, oral and maxillofacial medicine, and vascular surgery | pre-and-post observational study (descriptive statistics, Wilcoxon signed-rank test, multilevel linear regression, ANOVA) | *predictor(s)*: EHR implementation; *outcome(s)*: documentation time, time for patient care, combined patient care and documentation tasks | *task time*: documentation, patient care, peer communication, other activities; *consultation duration (minutes);* number of consultations per hour, mean duration; *time spent*: percentage of time spent, total mean time | No significant difference in consultation duration or number of consultations per hour post-implementation in either hospitals; post-implementation significant decrease in time spent dedicated to patient care at the legacy-EHR center; at paper-based center, significant increase in dedicated time spent on documentation, and significant decrease in time on combined patient care and documentation; significant difference in the effect of EHR implementation on dedicated documentation time between centers. | Cannot make any conclusions shifts of documentation tasks to distinct time periods, possible overestimation of consultation time (e.g., time spent handling telephone calls), small sample size, not controlled for patient characteristics, different distribution of specialists at each site, observation data |
| Kadish et al. (2018); USA | EMR usage reports | *effort*: time spent with EMR; time in system (TIS) after-hours; *unit(s)*: percentage TIS | Epic Systems | physicians, nurse practitioners and physician assistants (n=185); ambulatory; medical oncology | pre-and-post observational study (descriptive statistics, paired Wilcoxon test) | *predictor(s)*: individually tailored EMR training; *outcome(s)*: time spent on EMR activities | *provider survey* *(5-point Likert scale)*: overall EMR confidence in placement of orders, documentation, chemotherapy ordering, clinical review, and inbox message management; *individualized EMR utilization profile (efficiency in EMR use)*: active (30 seconds or less of inactivity) TIS for clinical review, documentation, inbox management, order placing, other, and after-hours (defined as 7:00pm-6:00am); *office visits closed same day*: percent documentation completed and charge entered | TIS decreased among most EMR activities; statistically significant reduction in TIS for documentation, placing orders, and other activity, and no difference in TIS after hours after training. | response bias, incomplete calculation of activities (occurring before, during, and after patient visit), no triangulation of data, self-reported data, observation data |
| Kannampallil et al. (2018); USA | EHR log files | *effort*: EHR use (documentation and review); *unit(s)*: time spent per patient (seconds) | Epic Systems | physicians (n=8); emergency department; emergency medicine | retrospective observational study (Pearson's correlation) | *predictor(s)*: EHR use; *outcome(s)*: ED performance metrics | *EHR log activities*: time spent on documentation, review, orders, and onscreen navigation; *performance metrics*: door-to-provider time, door-to-doctor time, door-to-disposition time, and length of stay (LOS); *patient acuity*: Emergency Severity Index | Statistically significant positive correlation was found between physician review of patient charts and door-to-disposition time, and between physician review of patient charts with LOS. | no causal effect, generalizability (attendings only), colinear performance metrics, small sample, patients with lower Emergency Severity Index only |
| Karp et al. (2019); USA | EHR system timers and event logs, video recording timers | *effort*: time spent on EHR tasks, number of clicks; *unit(s)*: average time spent (minutes per task), count (mean) | Cerner | nurses [n=536 admission patient histories (APHs (pre-intervention)), n=640 APHs (post-intervention)]; ambulatory, inpatient and emergency department; medical-surgical, ICU, emergency medicine, step-down and telemetry | experimental pre-and post nonrandomized prospective cohort design (descriptive statistics, independent samples t-test) | *predictor(s)*: admission patient history (APH) implementation; *outcome(s)*: quality and efficiency | *quality*: number and proportion of essential data elements captured, proportion of essential data elements completed in one sequence; *efficiency*: average time to complete admission patient history (minutes), number of mouse clicks to complete admission patient history (mean) | Statistically significant decrease in the average active time spent documenting the APH post-intervention; statistically significant decrease in the average number of clicks required to document the APH post-intervention also found. | generalizability (health systems with EHR timers and event logs), unable to capture intermittent documentation (measurement of uninterrupted documentation instances only) |
| Krawiec et al. (2019); USA | EHR timestamp data (Cerner Advance) | *effort*: EHR use; *unit(s)*: time spent per encounter (minutes) | Cerner | physicians (n=21); inpatient; emergency medicine, pediatrics and internal medicine | retrospective observational study (descriptive statistics, Kruskal–Wallis test) | *predictor(s)*: critical care type; *outcome(s)*: time spent in the EHR | *EHR activities*: total time spent in EHR (active EHR time), chart review time (time spent reviewing clinical documents, patient flowsheet and medication administration record), documentation time (time spent performing clinical documentation) and order entry (time spent performing orders) during pre-rounding (6:00am-8:30am); *patient acuity*: mechanical ventilator and vasoactive support presence or absence | EHR usage per patient encounter, time spent chart reviewing, time spent placing orders, and time spent documenting all significantly associated with critical care resources utilization. | no contextual information from EHR logs (e.g., direct patient care), software reports all EHR usage data within the time period, generalizability (Cerner, single institution, small sample size), retrospective observational study, could not link to patient care quality or outcomes, measurement error (e.g., EHR usage may have been overestimated in critical care context), vendor-defined active time |
| Krawiec et al. (2020); USA | EHR timestamp data (Cerner Advance) | *effort*: EHR usage after-hours; *unit(s)*: time spent per patient (seconds) | Cerner | physicians (n=7); inpatient; medical, general surgical and cardiothoracic pediatric ICU | retrospective observational study (descriptive statistics, Pearson's correlation, logistic regression) | *predictor(s)*: EHR documentation time (EHR after-hours); *outcome(s)*: patient census and mortality | *EHR activities*: total time spent in EHR (active EHR time), chart review time (time spent reviewing clinical documents, patient flowsheet and medication administration record), documentation time (time spent performing clinical documentation) and order entry (time spent performing orders) during pre-rounding; *time period*: regular hours (8:00am-7:00pm), all hours (8:00am-8:00am the next day), after-hours (7:00pm-8:00am); *patient census*: clinical notes written or attested per day attending was on service and in Virtual PICU Systems database; *patient acuity*: Pediatric Risk of Mortality (PRISM) 3 and Pediatric Index of Mortality (PIM) 2 scores; *time spent per patient*: sum of usage time over number of clinical notes signed per day | Higher patient consensus associated with significant increase in odds of EHR after-hour usage; admission PIM2 and PRISM3 scores not associated with increased odds of EHR after-hour usage. | generalizability (single institution, physicians), small sample size, measurement error (“after-hours” defined based on knowledge of PICU workflow), communication not captured, potential patient confounders (e.g., patient complexity, patient comorbidities, etc.), vendor-defined active time, no data on remote EHR access |
| Mamykina et al. (2012); USA | iPad activity capture tool | *effort*: task transition rate; *unit(s)*: average time per day (minutes), task transition rate per hour | Allscripts | physicians (n=11); ambulatory; general medicine | time-and-motion study, member checks (descriptive statistics) | *predictor(s)*: n/a; *outcome(s)*: task allocation time and task switching | *time spent on note writing daily*: time start to save/submit, *transitions*: frequencies and rates of common transitions between activities that were captured in sequence; *type of documenter*: early, thorough, opportunistic | High level of fragmentation in documentation activities and frequent task transitions associated with electronic documentation practices; three categories of documenters identified: early, thorough, and opportunistic; on average, physicians transitioned more than 10 times while note writing and referenced other resources at least 4 times on average; prior to documentation, physicians engaged in researching patient case and information gathering while post-documentation involved other tasks such as medication list updates. | generalizability (one unit of teaching hospital, limited number of participants, residents, one EHR system), pre-defined taxonomy where activities outside taxonomy captured as other, observer bias |
| Mamykina et al. (2016); USA | iPad application | *effort*: computer-based tasks vs. interacting with patients, multitasking; *unit(s)*: time spent per shift (minutes), proportion of total activity time | Allscripts | physicians (n=7); ambulatory; general medicine | time-and-motion study, informal interviews (member checks) (descriptive statistics, thematic analysis) | *predictor(s)*: n/a; *outcome(s)*: time spent on task categories | *clinician activities*: frequency, duration, average total time spent per task, proportion of average shift time spent per task, proportion of average total computer-based activities time spent per task; *multitasking*: time spent | Half of resident shift time was spent using computers while less than one-tenth was spent interacting with patients; nearly 60% of time spent multitasking; among computer-based activities, over half involved computer-based documentation activities including writing notes and reading notes. | generalizability (single institution, general medicine service only), small sample size, period of transition for interns |
| Marmor et al. (2018); USA | EHR system reports | *effort*: time spent logged into systems outside clinic hours; *unit(s)*: monthly average hours per clinic day | not specified | physicians (n=39); ambulatory; general medicine, cardiology and gastroenterology | longitudinal observational study (descriptive statistics, univariable regression, coefficient of determination) | *predictor(s)*: daytime and afterhours EHR usage; *outcome(s)*: likelihood to recommend provider score and communication domain score | *EHR usage*: average monthly hours logged into EHR during and outside normal clinic hours; average monthly hours of EHR usage per day of clinic; *patient satisfaction*: Clinician and Group Consumer Assessment of Healthcare Providers (CG CAHPS) and Systems survey performance reports per provider | On average, internists and specialists spent 3.4 hours and 0.79 hours per clinic day logged into the EHR during daytime and after clinic hours, respectively; internists spent most time logged in EHR afterhours per month followed by gastroenterologists; no association between EHR usage time afterhours and CG CAHPS scores; daytime hours spent logged into the EHR and CG CAHPS scores significantly and inversely related in four domains: provider showed patient respect, provider knew patient’s history, overall communication quality, and likelihood to recommend provider. | Confounders (no information on patient demographics or wait times), no information on provider EHR usage in the exam room to help better understand the relationship between EHR usage and patient satisfaction, small sample size, self-reported data |
| Micek et al. (2020); USA | EHR event logs and timestamps | *effort*: afterhours time; *unit(s)*: minutes per day/half-day | Epic Systems | physicians (n=34); ambulatory; primary care | observational cohort study with cross-sectional and retrospective data (descriptive statistics, ordinal regression, bivariate analyses using nonparametric testing (Spearman’s rho, Mann–Whitney U-test), sensitivity analyses) | *predictor(s)*: EHR time; *outcome(s)*: burnout | *electronic survey*: self-reported, widely-used single-item question on burnout (5-point scale); *sociodemographics*; *visit volume*: total number of visits; *EHR time*: weekday work-hours in-clinic time, weekday work-hours out-of-clinic time, weekday afterhours time, weekend/holiday afterhours time (workday defined as 7:30am-5:29pm, and morning as 7:30am-12:29pm and afternoon as 12:30pm-5:29pm for half-day sessions); *administrative records*: physician sex and clinical FTE | Greater total and in-clinic EHR time associated with higher levels of burnout; controlling for age, sex, cFTE, visit volume, and other EHR time categories, burnout significantly associated only with in-clinic EHR time; no significant relationship between burnout and after-hours EHR use. | generalizability (academic internal medicine physicians, one university-based practice setting, lower face-to-face time and patients-per-hour requirements compared with other organizations), small sample size, low response rate, validity (burnout measure with a single-item), EHR access logs cannot discriminate between active or idle time, possible misclassification (only included time spent logged into outpatient environment not total EHR work), EHR metric not subdivided into EHR task categories performed, self-reported data |
| Mishra et al. (2018); USA | event log timestamps | *effort*: time spent on in-person visits, time spent on EHR documentation after clinic hours, time to close encounters; *unit(s)*: minutes spent per physician (mean) | not specified | physicians (n=18); ambulatory; primary care | dual-balanced crossover study with 4 periods, 2 sequences, and 2 treatments (descriptive statistics, chi-squared test, independent two-tailed paired t-tests, multilevel logistic and multilevel linear regression models, random-effects model) | *predictor(s)*: scribes; *outcome(s)*: EHR documentation, patient interaction and job satisfaction | *clerical burden*: clerical work extending into personal time [non-clinic hours on weekdays and on weekends (4-level ordinal scale)]; *quality of patient-physician interaction*: perception of time spent on EHR and on direct patient interaction during a clinical visit (4-level ordinal scale), physician perception of association between scribe use and quality of patient interactions (5-point Likert scale); *work satisfaction* (5-point Likert scale); *patient satisfaction*: patient perception of time spent by PCP on direct interaction vs. computer (3-point Likert scale), quality of visit (5-point Likert scale); *EHR activity*: proportion of physician’s clinic unit spent on EHR*,* mean minutes spent per physician spent on in-person visits and EHR documentation during and after clinic hours; *workflow efficiency (event logs):* time required to close encounters | Scribed periods were significantly associated with reduced self-reported after-hours EHR documentation, higher patient interaction with patients, and less time documenting in the EHR during a visit. Scribe period was also significantly associated with timely completion of encounter documentation by the end of the next business day. There was no significant decrease in time spent logged into the EHR during off hours or in the proportion EHR activity in each PCP clinic unit. | small sample size, recall bias, participants may be more amenable to scribes, no qualitative data, self-reported data |
| Overhage et al. (2020); USA | EHR log files | *effort*: time spent after hours; *unit(s)*: average time spent per encounter (seconds) | Cerner | physicians [n=154,719 (physicians), n=98,211,891 (patient encounters)]; ambulatory; allergy and immunology, cardiology, critical care, endocrinology, family medicine, gastroenterology, gerontology, hematology and oncology, infectious diseases, internal medicine, nephrology, physical medicine and rehabilitation, preventive medicine, primary care, pulmonology, rheumatology and sports medicine | descriptive study, cross-sectional multi-institutional national analysis (descriptive statistics) | *predictor(s)*: n/a; *outcome(s)*: time spent on 13 EHR functions | *EHR clinical activities*: total and active time spent on 13 clinically focused EHR functions such as writing documentation, placing orders, reviewing historical notes, or reviewing clinical decision-support alerts (average per encounter); *active time*: mouse clicks (per minute), mouse movement (pixels or more per minute), and keystrokes (per minute); *after-hours EHR use*: time spent in EHR between 6:00pm-6:00am on weekdays and anytime on weekends; *time per encounter*: all active EHR time in each category divided by number of ambulatory completed encounters; *completed encounter*: physician signed note | On average, more than 16 minutes total active EHR time spent per encounter; over a tenth of this time was spent afterhours; of the 13 functions, chart review, documentation, and ordering were the most time consuming; distribution of time spent by providers using EHRs varied within specialty, but proportion of time spent on EHR functions was similar across specialties. | generalizability (single EHR), misclassification (not knowing physician schedules and may have overestimated time afterhours), high variation between providers (high variability within subspecialties), variability in active EHR time is greater when summarized by month versus day, unable to analyze the sources of variation (differences in clinical processes or software configurations) |
| Saag et al. (2019); USA | user action logs | *effort*: "work after work" time; *unit(s)*: average time spent per day (minutes) | Epic Systems | physicians (n=573); ambulatory; dermatology, medicine, mental health, neurology, obstetrics/gynecology, pediatrics 28, rehabilitation, surgery and other | longitudinal cohort study (least squares means of fixed effects, Tukey’s multiple comparisons test) | *predictor(s)*: number of days with appointments per week; *outcome(s)*: "work after work" | *"work after work"*: minutes logged into the EHR at any time on days without scheduled appointments, or minutes logged into the EHR outside of 30 min before or after a provider’s first and last scheduled appointment | Time spent working in EHR on days without appointments positively associated number of appointment days per week; time spent in EHR after hours on days with scheduled appointments also positively associated number of appointment days per week. | no triangulation with burnout data |
| Sinsky et al. (2016); USA | Work Observation Method by Activity Timing (WOMBAT) | *effort*: proportion of time spent on activities, self-reported afterhours work; *unit(s)*: proportion of time spent | Epic, Allscripts, athenahealth, Centricity, NextGen, SRS, eClinicalWorks | physicians (n=57); ambulatory; family medicine, internal medicine, cardiology and orthopedics | quantitative direct observational time-and-motion study, self-reported diary (descriptive statistics, interobserver reliability) | *predictor(s)*: n/a; *outcome(s)*: time spent on task categories | *work activity*: proportion of time spent on direct clinical face time, EHR and desk work, administrative tasks, and other tasks (divided by the total observation time) plus multitasking; *self-reported afterhours work*: total time, time in EHR on off-duty-evenings, on-call | Nearly half of physicians' total time was spent on EHR and desk work; over one-third of this time was spent on documentation and review tasks; physicians spent 37.0% of their time on EHR and desk work in examination room with patients; those with documentation support spent more time on direct clinical face time with patients compared to those without; among those who completed after-hours diaries, 1.5-2.2 hours spent on after-hours work per day on average; 59-69% total time spent on EHR tasks. | self-selection bias (high-performing practices, after-hours diary), generalizability to other settings, descriptive study design without statistical comparisons, Hawthorne effect, self-reported data, small sample size, confounding |
| Smith et al. (2018); USA | EMR activity log, real time measurement system (RTMS) timestamp | *effort*: EMR charting process; *unit(s)*: minutes spent per encounter (mean) | not specified | nurses [n=28 (observation days)]; inpatient; intensive care unit | time-and-motion study, observational study (descriptive statistics, hierarchical task analysis, ANOVA) | *predictor(s)*: SOFA score, CI score and work experience; *outcome(s)*: time spent charting | *EMR activities*: average process time of each task category specifically EMR documentation; *patient sickness level*: Sequential Organ Failure Assessment (SOFA) scores, Charlson Comorbidity Index (CI) scores; *nurse experience*: years worked in ICU | Higher experience associated with significantly higher average time spent on in-room assessment charting; low experience significantly associated with higher average time spent on out-of-room assessment charting; nurses with low CI score patients took significantly longer to chart compared to those with high CI score patients. | small sample size, gaps in the RTMS data logs, secondary analysis of data |
| Tai-Seale et al. (2017); USA | EHR access timestamp | *effort*: time spent on desktop medicine; *unit(s)*: mean hours per day | Epic Systems | physicians (n=471); ambulatory; internal and family medicine, and pediatrics | retrospective observational study (descriptive statistics, linear regression) | *predictor(s)*: physician characteristics; *outcome(s)*: time spent on face-to-face visits and time spent on desktop medicine | *EHR activity*: workstation-patient-physician combination time blocks, time spent performing tasks (e.g., face-to-face visit time, desktop medicine); *physician characteristics* | Physicians similar time on direct patient encounters and desktop medicine on average; over a third of the time spent on desktop medicine was spent writing progress notes; clinics that did not apply the National Committee for Quality Assurance (NCQA) recognition experienced less face-to-face time with patients and more time with desktop medicine. | imprecision in measuring time spent on some activities (e.g., idle time on the computer), time gaps were not allocated to any patient activity, no causal inferences on relationship between patient-centered care and time allocation, generalizability (early EHR adopter) |
| Tipping et al. (2010); USA | personal digital assistant WorkStudy+ Plus application | *effort*: proportion of time spent on activities; *unit(s)*: proportion of time spent | not specified | physicians (n=24); inpatient; n/a | time-and-motion study (interobserver reliability, t-tests, Wilcoxon two-samples test) | *predictor(s)*: patient volume; *outcome(s)*: time spent on task categories | *hospitalist data*: number of patients at beginning of care, number of patients discharged during the day, number of admissions; patient load: admissions plus number of patients at beginning of day; *hospitalist activities*: Percent of total duration of activities including multitasking | Hospitalists spent the most time interacting with the EHR of all activities representing over a third of their time; majority of time spent in the EHR was on documenting and more than half the documentation involved progress notes; one-fifth of time spent in EHR was spent reading/reviewing notes; multitasking observed in 16% of all time recorded; majority of these occurrences less than one minute long; above average patient numbers significantly associated with a decrease in time spent interacting with EHR, but physicians reported delaying documentation until later in evening or next day. | generalizability (single urban, academic hospital, focused on daytime and weekday activities only), potential measurement error (data collection using nonclinical research assistants), observation data |
| Tran et al. (2019); USA | Provider Efficiency Profile audit log | *effort*: time managing inbox, time in the EHR, time in the EHR after hours; *unit(s)*: time spent per appointment (minutes) | Epic Systems | physicians, nurse practitioners and physician assistants (n=107); ambulatory; family medicine, internal medicine and pediatrics | cross-sectional study (descriptive analytics, simple linear regression, Fisher’s Exact Test) | *predictor(s)*: burnout; *outcome(s)*: performance | *Mini-Z questionnaire*: burnout (single item), job satisfaction (5-point Likert scale), and work-related stress (5-point Likert scale); *clinical full-time equivalents (cFTE)*: dedicated clinical time; *PEP EHR activity*: time spent in the EHR after hours (total minutes in EHR between 7pm-7am on scheduled days, total minutes in EHR on unscheduled days); *number of appointments*: scheduled outpatient face-to-face appointments; *inbox messages for results review and patient calls*: number of messages received, number of messages incomplete at end of reporting period, average number of days until message is marked complete; *“Close Office Visits the Same Day” metric:* percentage of a clinical encounters that were closed on the same day of the visit (documentation management) | Nearly 40% of providers reported burnout; significant association between higher clinical FTE and self-reported burnout; one-third of time spent in EHR was on completing clinical documentation; clinicians reporting burnout dedicated more time in EHR after hours per appointment compared to same clinical FTE; burnout significantly associated with lower rates of same day chart completion; same day chart completion rates were negatively associated time spent in EHR overall and also after hours; those who reported burnout received higher volume of inbox messages; burnout significantly associated with longer completion time for inbox messages and more incomplete messages. | small sample size, generalizability (academic faculty, outpatient setting), granularity of PEP metrics, confounders (on-call responsibilities, inbox coverage), self-reported clinical FTE, vender-defined active time |
| Wang et al. (2019); USA | EHR event log timestamps | *effort*: remote EHR actions; *unit(s)*: mean and proportion time spent per day (hours), median percent of EHR actions | Epic Systems | physicians (n=101); ambulatory; internal medicine | observational cohort study (descriptive statistics, Welch’s t-test, sensitivity analysis) | *predictor(s)*: rotations and roles; *outcome(s)*: time spent on task categories | *EHR usage (over a 24-hour cycle):* mean number of EHR actions logged per user day in half hour time intervals, and mean time spent on common EHR action categories per user-day (accounting for idleness) including chart review, note review, results review, note entry, order entry, and navigator use, mean daily EHR usage, median EHR actions, median proportion EHR actions accessed remotely; *median patient records accessed*; *rotation schedules* | PGY1 interns spent significantly more time on note entry compared to PGY2+ residents during both general medicine and emergency medicine rotations; PGY2+ residents spent significantly more time on note review compared to PGY1 interns during general medicine, emergency medicine and night team rotations. | estimation of idle time in access logs, single academic center |
| Zheng et al. (2010); USA | time-and-motion timestamp data | *effort*: workflow fragmentation, task switching; *unit(s)*: average continuous time (ACT [seconds]), proportion of time | Eclipsys | physicians [n=4 providers (pre-implementation), n=20 observations (pre-implementation), n=12 provider (post-implementation), n=22 observations (post-implementation)]; inpatient; pediatric intensive care unit | empirical validation study using the time and motion approach (descriptive statistics) | *predictor(s)*: n/a; *outcome(s)*: n/a | *workflow quantifier (magnitude of workflow fragmentation):* average continuous time (ACT) continuously spent on performing a single clinical activity or task category; *workflow fragmentation (task switching frequency)*: task switch rate; *consecutive sequential pattern analysis (CSPA)*: hourly occurrence rate of recurring workflow segments within and across observations*; transition probability analysis (TPA)*: transition probabilities of task pairs (total times transition observed divided by total number of transitions); *time allocation*: average aggregated clinician time (proportion) | Post-implementation significant decrease in the average amount of time continuously spent performing a single task, in particular, "computer--read", "personal" and "talking/rounding"; significant decrease in task duration in "direct patient care" and "miscellaneous"; hourly occurrence rate of transitions doubled or increased by more for "talking/rounding" to "walking/moving", "walking/moving" to "talking/rounding", "talking/rounding" to "computer--read" and "computer--read" to "talking/rounding"; Post-implementation only "talking/rounding" to "computer--read" had significant increase in transition probability, and significant decrease in the transition probability of "talking/rounding" to "paper--writing". | convenience sample of resident physicians, small sample size (unbalanced characteristics), generalizability (practice inferences, unique PICU setting, one CPOE system), observer bias, difficult to observe multitasking |
